# Insight into the heat transfer of third-grade micropolar fluid over an exponentially stretched surface

**DOI:** 10.1038/s41598-022-19124-5

**Published:** 2022-09-16

**Authors:** Kamel Guedri, N. Ameer Ahammad, Sohail Nadeem, ElSayed M. Tag-ElDin, Aziz Ullah Awan, Mansour F. Yassen

**Affiliations:** 1 Mechanical Engineering Department, College of Engineering and Islamic Architecture, Umm Al-Qura University, P.O. Box 5555, Makkah, 21955 Saudi Arabia; 2 Department of Mathematics, Faculty of Science, University of Tabuk, P.O. Box 741, Tabuk, 71491 Saudi Arabia; 3 Department of Mathematics, Quaid-i-Azam University, Islamabad, 45320 Pakistan; 4 Faculty of Engineering and Technology, Future University in Egypt, New Cairo, 11835 Egypt; 5 Department of Mathematics, University of the Punjab, Lahore, 54590 Pakistan; 6 Department of Mathematics, College of Science and Humanities in Al-Aflaj, Prince Sattam Bin Abdulaziz University, Al-Aflaj, 11912 Saudi Arabia; 7 Department of Mathematics, Faculty of Science, Damietta University, New Damietta, 34517 Damietta Egypt

**Keywords:** Engineering, Mathematics and computing

## Abstract

Due to their unique microstructures, micropolar fluids have attracted enormous attention for their industrial applications, including convective heat and mass transfer polymer production and rigid and random cooling particles of metallic sheets. The thermodynamical demonstration is an integral asset for anticipating the ideal softening of heat transfer. This is because there is a decent connection between mathematical and scientific heat transfers through thermodynamic anticipated outcomes. A model is developed under the micropolar stream of a non-Newtonian (3rd grade) liquid in light of specific presumptions. Such a model is dealt with by summoning likeness answers for administering conditions. The acquired arrangement of nonlinear conditions is mathematically settled using the fourth-fifth order Runge-Kutta-Fehlberg strategy. The outcomes of recognized boundaries on liquid streams are investigated in subtleties through the sketched realistic images. Actual amounts like Nusselt number, Sherwood number, and skin-part coefficient are explored mathematically by tables. It is observed that the velocity distribution boosts for larger values of any of $\alpha _1$, $\beta$, and declines for larger $\alpha _2$ and Hartmann numbers. Furthermore, the temperature distribution $\theta (\eta )$ shows direct behavior with the radiation parameter and Eckert number, while, opposite behavior with *Pr*, and *K*. Moreover, the concentration distribution shows diminishing behavior as we put the higher value of the Brownian motion number.

## Introduction

Many researchers around the globe are showing a keen interest in learning more about the non-Newtonian fluid flow. The motivation for studying these fluids is their potential usage in industries and technology. These fluids have paramount prominence in material processing, bioengineering, geophysics, oil reservoir engineering, chemical, nuclear, and many other fields. Many materials of our daily life use, such as apple sauce, mud, paints, shampoos, soaps, ice cream, condensed milk, polymeric liquids, low shear rate blood, pasta, oils, etc., show a highly complex nature and are taken as non-Newtonian liquids. Rheological physical attributes of a non-Newtonian fluid are impossible to explore by using the simple Navier-Stokes theory (unlike for viscid fluids). The third-grade fluid is one of the categories of differential type fluids. It describes shear thinning and thickening properties. Abbasbandy et al.^1^ analyzed both exact and series solutions for third-grade fluid using thin film. Hayat et al.^2^ scrutinized rotating third-grade MHD fluid flow bounded between two permeable sheets. Farooq et al.^3^ probed mass and thermic transmission for third-grade fluid inside a vertically positioned flow configuration besides viscous dissipation. Sinha^4^ studied MHD third-grade fluid flow inside a pipe having stretched and porous walls. Okoya^5^ examined the heat-exchange properties of an exothermic reactive third-grade fluid across a circular passage by considering the Reynolds viscosity model and its uses in the processing industries. Hayat et al.^6^ explored MHD third-grade fluid flow bounded with convective heated and stretched surfaces. Magnetohydrodynamic stagnation point flow for non-Newtonian (third-grade) fluid caused by the non-linearly stretchy surface is examined by Hayat et al.^7^. Interested readers can see some recent work on third-grade fluids here^8–11^.

Eringen^12^ was the pioneer who studied the flow of micropolar fluids. Physical examples of micropolar fluids can be seen in ferrofluids, blood flows, bubbly liquids, liquid crystals, and so on, all of them containing intrinsic polarities. Eringen’s hypothesis^12^ proclaims that any fluid formed from rigid, arbitrarily oriented, or spherical particles with particular micro-rotations is called the micropolar fluid. Guram and Smith^13^ studied flow subject to stagnation point for a micropolar liquid model. The flow model of a micropolar liquid caused by a heat source over an axisymmetric and rotating sheet is probed by Gorla and Takhar^14^. By considering combined convective conditions, Gorla et al.^15^ presented an axisymmetric stagnation point stream through a perpendicular standing cylinder for micropolar fluid. MHD effects on micropolar liquid stream across a continually moving plate were investigated by Sadeek^16^. Eldahab et al.^17^ probed the influence of radiations on thermal transmission for a micropolar liquid stream across a flat sheet in a permeable medium. Mohamed and Abo-Dahab^18^ deliberated the consequences of thermal radiations and chemical reactions on mass and thermal transport for micropolar fluid in penetrable media. Investigation for the micropolar fluid flow due to permeable stretched sheet also consequent thermal transmission is done by Turkyilmazoglu^19^. The darcy-Forchheimer flow of 3D micropolar fluid along horizontal parallel sheets in the revolving system in a penetrable medium is analyzed by Khan et al.^20^. For an in-depth investigation of micropolar fluid flow, we refer the curious reader to some recent studies^21–25^.

Over the decade, stream due to the extendable sheet has acquired astonishing significance among scientists due to its modern design execution. Not many of these applications incorporate hot rolling, paper production, glass blowing, polymer and metal expulsion, and precious stone development. Crane^26^ introduced the investigation of stream subject to an extended sheet. Brady and Acrivos^27^ concentrated on the liquid stream in an extended channel. Specialists observed that an answer for a particular worth of Reynolds number exists for a 2D stream. Ahmad et al.^28^ performed the computational investigation of an unstable (3-D) chemically reacting MHD flow of Maxwell fluid. Ahmad et al.^29^ used the Cattaneo-Christov thermal influx model to probe the hybrid Micropolar nanoliquid stream with triple stratification. Many researchers have investigated heat and thermal investigation of fluids over stretched surfaces^30–36^. Khan et al.^37^ explored the MHD stream of second-grade fluid with multiple slip constraints. The non-Newtonian fluid has been widely discussed by^38–42^.

The above-cited literature review shows that little attention is paid to the study considering the effects of third-grade micropolar fluid flow over an expanding surface. Motivated by the numerous uses of non-Newtonian fluids and nanofluids^43–49^, a third-grade micropolar liquid stream subject to a stretching sheet is investigated here. The primary purpose of this extensive study is to improve thermal transportation subject to the existence of micro-rotations of tiny nanoparticles. The numerical plan is made in the approaching segment by utilizing liquid stream presumptions. With the utilization of likeness changes, an arrangement of PDEs is decreased into a non-straight arrangement of ODEs. The mathematical strategy RK45 is used to settle the acquired framework systematically. The influence of some pertinent parameters is spotlighted on each temperature field $\theta (\eta )$, concentration field $\phi (\eta )$, velocity filed $f'(\eta )$, and micropolar field $h(\eta )$ through graphical representations Figs. 2, 3, 4, 5, 6, 7, 8, 9, 10, 11, 12, 13. Tables 1, 2, 3 help understand the numeric variance of skin-friction coefficient, Nusselt and Sherwood number caused by involved parameters. This report is related to various applications in manufacturing plastic and metal spinning, paints, liquids material, crude oil purification, heat exchangers, polymer extrusion, etc.

## Flow analysis

**Figure 1:**
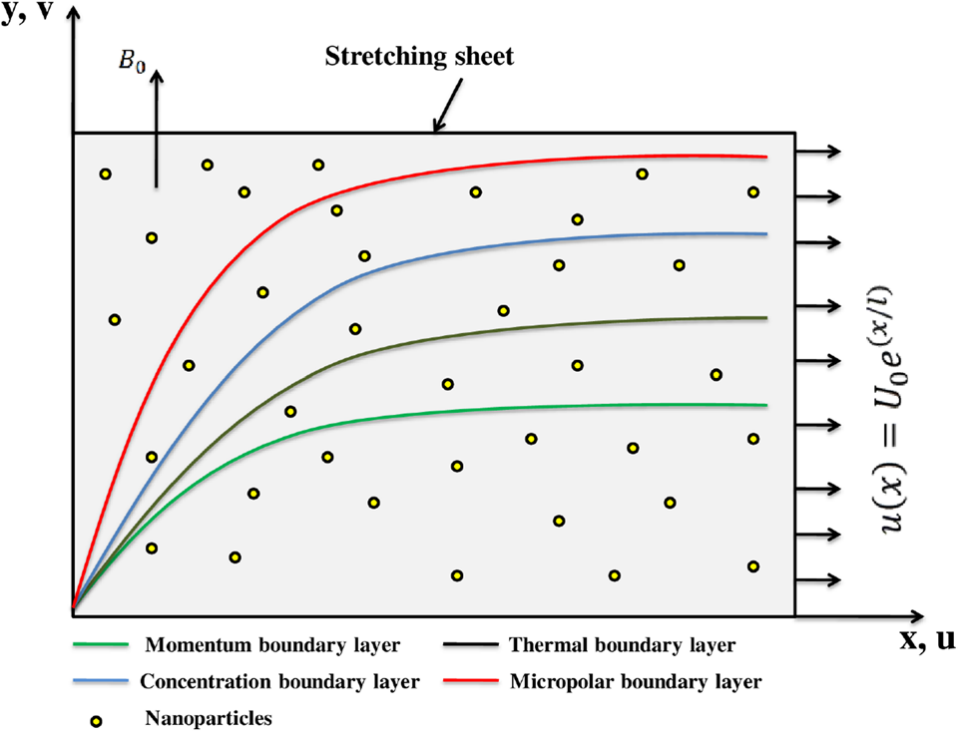
Flow geometry.

Steady incompressible and 2D stream of micropolar liquid over an extendable sheet is to be investigated in this communication. The *x*-axis is supposed along the continuously expanded surface while *y*-axis is taken perpendicular to this assumed surface in upward direction to the fluid (See Fig. 1). For the third grade fluid, the stress tensor is as follows:

$$\begin{aligned} {\mathbf {T}}=-p{\mathbf {I}}+\mu \mathbf {B}_{\mathbf{1}}+ {\alpha ^{*}_1}\mathbf {B}_{\mathbf{2}}+{\alpha ^{*}_2} {\mathbf {B}_{{\mathbf{1}}^{\mathbf{2}}}}+{\beta _1}\mathbf {B}_{\mathbf{3}} +{\beta _2}(\mathbf {B}_{\mathbf{1}}\mathbf {B}_{\mathbf{2}}+\mathbf {B}_{\mathbf{2}} \mathbf {B}_{\mathbf{1}})+{\beta _3}\mathbf {B}_{\mathbf{1}}(trc{\mathbf {B}_{\mathbf{1}}^{\mathbf{2}}}). \end{aligned}$$

Here *p*, ${\mathbf {I}}$, ${\mathbf {T}}$, and ${\mathbf {S}}$ are the pressure, identity tensor, Cauchy stress tensor, and the extra stress tensor respectively. Furthermore, ${\alpha ^{*}_k}(k=1,2) ,\, {\beta _j}(j=1,2,3)$ are metallic constants and $\mathbf {B}_{\mathbf{i}}(i=1,2,3)$ are the kinematic tensors defines as:

$$\begin{aligned} \mathbf {B}_{\mathbf{1}}&=({\mathbf {L}})^T+{\mathbf {L}},\\ \mathbf {B}_{\mathbf{n}}&=\frac{D\mathbf {B}_{\mathbf{n}-\mathbf{1}}}{Dt} +\mathbf {B}_{\mathbf{n}-\mathbf{1}}{\mathbf {L}}+({\mathbf {L}})^{T}\mathbf {B}_{\mathbf{n}-{\mathbf{1}}},\;\;\;\;\; n=2,3\\ \text {where},\;\;\;\;\;\;{\mathbf {L}}&=\nabla {{\mathbf {V}}}. \end{aligned}$$

Using the boundary layer estimations^50–53^ in case of 3rd-grade fluid, notably, inside boundary layer $\frac{\partial p}{\partial x}, \frac{\partial ^2 u}{\partial x}, \frac{\partial u}{\partial x}$, and *u* are *O*(1), *v* and *y* are $O(\delta )$, $\frac{\hat{\alpha _j}}{\rho }(j=1,2)$ and $\nu$ be $O(\delta ^2)$, and $\frac{\hat{\beta _k}}{\rho }(k=1,2,3)$ being $O(\delta ^4)$ as well as the components of $O(\delta )$ are ignored ($\delta$ is boundary layer width), the mathematical model for the said flow is as follows:

1 $$\begin{aligned} \frac{\partial v}{\partial y}+\frac{\partial u}{\partial x}=0, \end{aligned}$$

2 $$\begin{aligned} v\frac{\partial u}{\partial y}+u\frac{\partial u}{\partial x}= & {} \frac{\alpha ^{*}_{1}}{\rho }\left( \frac{\partial u}{\partial x}\frac{\partial ^2 u}{\partial y^2}+u\frac{\partial ^3 u}{\partial x \partial y^2}+3\frac{\partial u}{\partial y}\frac{\partial ^2 v}{\partial y^2}+v\frac{\partial ^3 u}{\partial y^3}\right) +2\frac{\alpha ^{*}_{2}}{\rho }\frac{\partial u}{\partial y}\frac{\partial ^2 v}{\partial y^2}\nonumber \\&+6\frac{\beta _3}{\rho }{\left( \frac{\partial u}{\partial y}\right) }^2\frac{\partial ^2 u}{\partial y^2}+\left( \nu +\frac{k}{\rho }\right) \frac{\partial ^2 u}{\partial y^2}+\frac{k}{\rho }\frac{\partial N}{\partial y}-\frac{\sigma B^{2}_{0}}{\rho }u, \end{aligned}$$

3 $$\begin{aligned} v\frac{\partial T}{\partial y}+u\frac{\partial T}{\partial x}= & {} \frac{(\mu +k)}{(\rho C_p)_f}{\left( \frac{\partial u}{\partial y}\right) }^2+\frac{Q}{(\rho C_p)_f}\left( T-T_{\infty }\right) -\frac{1}{(\rho C_p)_f}\frac{\partial q_r}{\partial y}+\frac{1}{(\rho C_p)_f}\frac{\partial }{\partial y}\left( k_1\left( T\right) \frac{\partial T}{\partial y}\right) \nonumber \\&+\tau \left( \frac{D_T}{T_\infty }{\left( \frac{\partial T}{\partial y}\right) }^2+D_B\frac{\partial T}{\partial y} \frac{\partial C}{\partial y}\right) , \end{aligned}$$

4 $$u\frac{{\partial \widehat{C}}}{{\partial x}} + v\frac{{\partial \widehat{C}}}{{\partial y}} = D_{B} \frac{{\partial ^{2} \widehat{C}}}{{\partial y^{2} }} + \left( {\frac{{\partial ^{2} T}}{{\partial y^{2} }}} \right)\frac{{D_{T} }}{{T_{\infty } }},$$

5 $$u\frac{{\partial \widehat{N}}}{{\partial x}} + v\frac{{\partial \widehat{N}}}{{\partial y}} = \frac{{\partial ^{2} \hat{N}}}{{\partial y^{2} }}\left( {\frac{\gamma }{{\rho j}}} \right) - \frac{k}{{j\rho }}\left( {2\widehat{N} + \frac{{\partial u}}{{\partial y}}} \right),$$

subjected to the boundary conditions

6 $$\begin{aligned} {\left\{ \begin{array}{ll} N=-m\frac{\partial u}{\partial y},\,\,\,\,\,\,\,\,\,\,u=U_0\,exp(x/l),\,\,\,\,\,\,T_{f}+h_f\left( \frac{\partial T}{\partial y}\right) =T,\,\,\,\,\,\,\,\,v=0,\,\,\,\,\,\frac{D_T}{T_\infty }\frac{\partial T}{\partial y}+D_B\frac{\partial C}{\partial y}=0,\,\,\,\,\,\,\text {as} \,\,\,\,y=0,\\ N \rightarrow 0,\,\,\,\,\,\,\,\,{u \rightarrow 0},\,\,\,\,\,\,\,\, \widehat{C} \rightarrow \widehat{C}_\infty ,\,\,\,\,\,\,\,\,T \rightarrow T_\infty ,\,\,\,\,\,\,\,\,\,\,\,\text {as} \,\,\,\,y\rightarrow \infty , \end{array}\right. } \end{aligned}$$

where *v* and *u* are the parts of velocity in *y* and *x* directions respectively. $\widehat{C}$ denotes concentration, *N* being Part of microrotation vector, orthogonal to the considered plane, and $\rho$ is density of fluid. $D_T$ shows thermophoresis diffusion coefficient, $D_B$ is Brownian diffusion coefficient, and $\tau =\frac{(\rho C_p)_{nf}}{(\rho C_p)_f}$ is the quotient of the heat capacity of the nano-particles and the heat of base fluid. $C_\infty$ and $T_\infty$ are free stream concentration and temperature. $\mu$ denotes dynamic viscosity whereas $\nu =\frac{\mu }{\rho }$ represents kinematic viscosity, $C_p$ is specific heat, *T* for temperature, $\gamma$ shows spin gradient viscosity, $k_1(T)$ is variable thermal conductivity, *j* is microinertia density, $k_f$ is thermal conductivity of base fluid, *k* is the vortex viscosity, and $(\alpha _1^*, \alpha _2^*, \beta _3)$ are material constants. Taking $\gamma$ as

7 $$\begin{aligned} \gamma =\left( \frac{k}{2}+\mu \right) j=\left( 1+\frac{K}{2}\right) \mu j, \end{aligned}$$

and define $K=\frac{k}{\mu }$ as micropolar parameter.

Also, suppose that

8 $$\begin{aligned} k_1\left( T\right) =k_f\left( 1+\epsilon \theta \left( \eta \right) \right) . \end{aligned}$$

The Rosseland radiative heat flux is $q_r=\frac{-4\sigma ^*}{3k^*}\frac{\partial ^2 T}{\partial y^2}\approx 4T^{3}_{\infty }T-3T^{4}_{\infty }$, where mean absorption coefficient is shown as $k^*$ and $\sigma ^*$ represents Stefan-Boltzmann constant.

9 $$\begin{aligned} \frac{\partial q_r}{\partial y}=\frac{-16T^{3}_{\infty }\sigma ^*}{3k^*}\frac{\partial ^2 T}{\partial y^2}. \end{aligned}$$

Equation (3) is now transformed as

10 $$\begin{aligned} v\frac{\partial T}{\partial y}+u\frac{\partial T}{\partial x}= & {} \frac{(k+\mu )}{(C_p \rho )_f}{\left( \frac{\partial u}{\partial y}\right) }^2+\frac{1}{(\rho C_p)_f}\frac{16T^{3}_{\infty }\sigma ^*}{3k^*}\frac{\partial ^2 T}{\partial y^2}+\tau \left( D_B\frac{\partial C}{\partial y}\frac{\partial T}{\partial y}+{\left( \frac{\partial T}{\partial y}\right) }^2\frac{D_T}{T_\infty }\right) \nonumber \\&+\frac{1}{(\rho C_p)_f}\frac{\partial }{\partial y}\left( \frac{\partial T}{\partial y}k_1\left( T\right) \right) +\frac{Q}{(\rho C_p)_f}\left( T-T_{\infty }\right) . \end{aligned}$$

Defining the similarity variables   $h, \,\,\, f,\,\,\, \eta ,\,\,\, \text {and}\,\,\, \theta$ as:

11 $$\begin{aligned} {\left\{ \begin{array}{ll} \psi \left( x,y\right) =\sqrt{2\nu l U_0}exp\left( \frac{x}{2l}\right) f\left( \eta \right) ,\,\,\,\,N=U_0 \sqrt{\frac{U_0}{2\nu l}}exp\left( \frac{3x}{2l}\right) h\left( \eta \right) ,\\ \phi (\eta )=\frac{C-C_\infty }{C_f-C_\infty },\,\,\,\,\theta (\eta )=\frac{T-T_\infty }{T_f-T_\infty },\,\,\,\, \eta =\sqrt{\frac{U_0}{2\nu l}}exp\left( \frac{x}{2l}\right) y, \end{array}\right. } \end{aligned}$$

where $\psi \left( x,y\right)$ is the stream function.

$$\begin{aligned} u=\frac{\partial \psi }{\partial y}= U_0 exp\frac{x}{l}f'\left( \eta \right) ,\,\,\,\,\,\,\, \texttt {and}\,\,\,\,\,\,\, v=-\frac{\partial \psi }{\partial x}=-\sqrt{\frac{\nu U_0}{2l}} exp\frac{x}{2l}\left( f+\eta f'\right) , \end{aligned}$$

$\eta$ is similar variable, while $\theta (\eta )$, $f'(\eta )$ and $\phi (\eta )$ are the similarity representation of temperature, velocity profile, and concentration. $h(\eta )$ is dimensionless micropolar profile respectively. Furthermore, $T_f$ and $C_f$ represent the temperature and concentration at the wall of the sheet, respectively. Mass conservation equation is automatically satisfied by putting Eq. (11) in Eq. (1). The PDEs (2), (4), (5), and (10) are transmuted to the non-linear coupled ODEs:

12 $$\begin{aligned}{}&(1+K)f'''+f''f+\alpha _1\left[ -ff^{(iv)}-2\eta f''' f''-9\left( f''\right) ^2+3f'''f'\right] -\alpha _2\left[ 3\left( f''\right) ^2+\eta f'''f''\right]\nonumber \\&\qquad +3\beta \left( f''\right) ^2f'''-2Ha^2 f'-2\left( f'\right) ^2=0, \end{aligned}$$

13 $$\begin{aligned}{}&\frac{1}{Pr}\left[ \epsilon \left( \theta '\right) ^2+\theta ''+\epsilon \theta \theta ''\right] +f\theta '+\left( 1+K\right) Ec\left( f''\right) ^2+\frac{4}{3} Rd\theta ''+\delta \theta +\left( \theta '\right) ^2 Nt+\theta '\phi 'Nb=0, \end{aligned}$$

14 $$\left( {\frac{{Nt}}{{Nb}}} \right)\theta ^{\prime\prime} + \phi ^{\prime\prime} + Lef\phi ^{\prime} = 0,$$

15 $$\begin{aligned}{}&fh'+\left( 1+\frac{K}{2}\right) h''-3hf'-2KB\left( f''+2h\right) =0. \end{aligned}$$

With the altered BCs as:

16 $$\begin{aligned} {\left\{ \begin{array}{ll} f'(\infty )=0,\,\,\,\,f''(\infty )=0,\,\,\,\,f(0)=0,\,\,\,\,f'(0)=1,\\ h(\infty )=0,\,\,\,\,\,\,\,\,\,\phi (\infty )=0,\,\,\,\,\,\,\,\,\,\theta '(0)Nt+\phi '(0)Nb=0,\\ \theta (\infty )=0,\,\,\,\,\,\theta (0)=1+\delta _T \theta '(0),\,\,\,\,\,h(0)=-mf''(0). \end{array}\right. } \end{aligned}$$

Here involved dimensionless variables are given as

17 $$\begin{aligned} {\left\{ \begin{array}{ll} \alpha _1=\frac{U_0}{\rho \nu l}\alpha _1^* exp\left( \frac{x}{l}\right) ,\,\,\,\,\,\,\,\,\,\,\,\,\,\,\,\,\alpha _2=\frac{U_0}{\rho \nu l}\alpha _2^* exp\left( \frac{x}{l}\right) ,\,\,\,\,\,\,\,\,\,\,\,\,\,\,\,\,\beta =\frac{U_0^3}{\rho \nu ^2 l}\beta _3 exp\left( \frac{3x}{l}\right) ,\,\,\,\,\,\,\,\,\,\,\,\,\,\,\,\,Ha^2=\frac{\sigma B_0^2 l}{\rho U_0}\,exp(-x/l),\\ Pr=\frac{\mu C_p}{k_f},\,\,\,\,\,\,\,\,\,\,\,\,\,\,\,\,\,\,\,\,\,\,\,\,\,\,\,\,\,\,\,\,\,\,\,\,\,\,\,K=\frac{k}{\mu },\,\,\,\,\,\,\,\,\,\,\,\,\,\,\,\,\,\,\,\,\,\,\,\,\,\,\,\,\,\,\,\,\,\,\,\,\,\,\,\,\,\,\,\,Nb=\frac{\tau D_B}{\nu }\left( \widehat{C}_f-\widehat{C}_\infty \right) ,\,\,\,\,\,\,\,\,\,\,\,\,Le=\frac{\nu }{D_B},\\ Nt=\frac{\tau D_t}{\nu T_\infty }\left( T_f-T_\infty \right) ,\,\,\,\,\,\,\,\,\,\,B=\frac{\nu l}{U_0 j}\,exp(-x/l),\,\,\,\,\,\,\,\,\,\,\,\,\delta =\frac{2lQ}{U_0} exp\left( \frac{l}{x}\right) ,\,\,\,\,\,\,\,\,\,\,\,\,\,\,\,\,\,\,\,\,\,\,\,\,\,\,\,Rd=\frac{4T_\infty ^3 \sigma ^*}{\mu k^* C_p},\\ Ec=\frac{U_0^2}{C_p\left( T_f-T_\infty \right) }exp\left( \frac{2x}{l}\right) ,\,\,\,\,\,\,\,\,\,\,\,\,\, \delta _T=h_f \sqrt{\frac{U_0}{2 \nu l}}exp \left( \frac{x}{2l}\right) , \end{array}\right. } \end{aligned}$$

where $\delta _T$ is thermal slip, *B* is microinertia density parameter,*Ec*, *Pr*, $Ha^2$ and *Le* are Eckert, Prandtl, Hartmann, and Lewis number respectively, *Nt* is Thermophoresis parameter, *Nb* is Brownian motion parameter, *K* the micropolar parameter, $\delta$ is the heat generation/absorption parameter, *Rd* the radiation parameter, $\beta$, $\alpha _1$ and $\alpha _2$ are 3rd grade, cross viscous and viscoelastic parameter.

The local Sherwood number $Sh_x$, skin friction coefficient $Cf_x$ and local Nusselt number $Nu_x$ are

18 $$\begin{aligned} Sh_x=\frac{xj_w}{D_B\left( \widehat{C}_f -\widehat{C}_\infty \right) },\,\,\,\,\,\,\,\,\,\,\,\,\,\,\,\,\,Cf_x=\frac{\tau _w}{\rho U_w^2},\,\,\,\,\,\,\,\,\,\,\,\,\,\,\,\,\,Nu_x=\frac{xq_w}{k_f\left( T_f -T_\infty \right) }, \end{aligned}$$

where

19 $$\begin{aligned} j_w&=-D_B\left. \left( \frac{\partial C}{\partial y}\right) \right| _{y=0}, \end{aligned}$$

20 $$\tau _{w} = \left[ {k\hat{N} + \left( {k + \mu } \right)\frac{{\partial u}}{{\partial y}} + 2\beta _{3} \left( {\frac{{\partial u}}{{\partial y}}} \right)^{3} + \alpha _{1}^{*} \left( {v\frac{{\partial ^{2} u}}{{\partial y^{2} }} + u\frac{{\partial ^{2} u}}{{\partial y\partial x}} + 2\frac{{\partial u}}{{\partial x}}\frac{{\partial u}}{{\partial y}}} \right)} \right]_{{y = 0}},$$

21 $$\begin{aligned} q_w&=-\left( k_f+\frac{16T^{3}_{\infty }\sigma ^*}{3k^*}\right) \left. \frac{\partial T}{\partial y} \right| _{y=0}. \end{aligned}$$

The above expressions in the form of similarity variables are:

22 $$\begin{aligned} \frac{1}{\sqrt{2}}\sqrt{Re_x}Cf_x&=\left[ \left( K+1\right) f''(\eta )+\alpha _1\left( \frac{7}{2}f''(\eta )f'(\eta )-\frac{1}{2}f(\eta )f'''(\eta )\right) +\beta \left( f''(\eta )\right) ^3+Kh(\eta )\right] _{\eta =0}, \end{aligned}$$

23 $$\begin{aligned} \sqrt{\frac{2}{X}}\frac{Nu_x}{\sqrt{Re_x}}&=-\left( 1+\frac{4}{3} Rd\right) \theta '(0), \end{aligned}$$

24 $$\begin{aligned} \sqrt{\frac{2}{X}}\frac{Sh_x}{\sqrt{Re_x}}&=-\phi '(0), \end{aligned}$$

where $X=\frac{x}{l}$ and $Re_x=\frac{U_w(x)x}{\nu }$ denotes the local Reynolds number.

## Solution of the problem

The PDEs system describing the above model is transmuted into an ODEs system by using suitable similarity variables. These ODEs system is handled by Runge-Kutta 4th order (RK-4) numerical method. Generally RK-4 method is most familiar. It is easy to implement, self-starting, and very stable method. The numerical solution for the problem is done by taking fixed values of similar variables $\epsilon =0.3$, $Pr=1.5$, $K=0.2$, $Ec=0.8$, $\delta =0.5$, $Nt=0.3$, $Rd=0.5$,, $B=1.0$, $\delta _T=0.8$, $m=0.5$,$Nb=1.0$, and $Le=0.7$. This estimation is needed to provide one of the solution for PDEs, otherwise the PDEs are very difficult to tackle. The procedure for solving the problem is

$$\begin{gathered} p_{1} = f,\;p_{2} = f^{\prime},\;\;p_{3} = f^{\prime\prime},\;p_{4} = f^{\prime\prime\prime},\;pp_{1} = f\left( ^{\textit{iv}} \right),p_{5} = \theta ,\;p_{6} = \theta ^{\prime} \hfill \\ pp_{2} = \theta ^{\prime\prime},\;p_{7} = \phi ,\;p_{8} = \phi ^{\prime} \quad pp_{3} = \phi ^{\prime\prime},\;p_{9} = h,\;p_{{10}} = h^{\prime},\;pp_{4} = h^{\prime\prime}. \hfill \\ \end{gathered}$$

The transformed ODEs are

$$\begin{gathered} pp1 = (\alpha _{1} *p_{1} )^{{ - 1}} *\left( { - 2*p_{2} + \left( {1 + K} \right)*p_{4} + p_{1} *p_{3} + 3*\alpha _{1} *p_{2} *p_{4} + \left( { - 9*\alpha _{1} - 3*\alpha _{2} } \right)*p_{3} *p_{3} } \right. \hfill \\ \quad \quad \left. { - \left( {2*\alpha _{1} + \alpha _{2} } \right)*\eta *p_{3} *p_{4} + 3*p_{3} *p_{3} *p_{4} *\beta - 2*Ha_{2} *p_{2} } \right), \hfill \\ pp2 = ( - 3*Pr)*(3 + 3* \in *p_{5} + 4*Rd*Pr)^{{ - 1}} *\left( {\left( {1 + K} \right)*Ec*p_{3} *p_{3} + p_{1} *p_{6} + \delta *p_{5} } \right. \hfill \\ \left. {\quad \quad + Nb*p_{8} *p_{6} + Nt*p_{6} *p_{6} + \frac{ \in }{{Pr}}*p_{6} *p_{6} } \right) \hfill \\ pp3 = \frac{{ - Nt}}{{Nb}}*pp2 - Le*p_{1} *p_{8} , \hfill \\ pp4 = 2*(2 + K)^{{ - 1}} *\left( {4*K*B*p_{9} + 2*K*B*p_{3} + 3*p_{2} *p_{9} - p_{1} *p_{{10}} } \right), \hfill \\ \end{gathered}$$

subject to the boundary constraints

$$\left\{ \begin{gathered} p_{2} = 1,\quad p_{1} = 0,\quad p_{5} + \delta _{T} * p_{6} ,\quad Nb * p_{8} + Nt * p_{6} = 0,\quad p_{9} = - m*p_{3} ,\quad {\text{as}}\;\;\eta \to {\text{0,}} \hfill \\ {\text{p}}_{{\text{9}}} = 0,\quad p_{3} = 0,\quad p_{2} = 0,\quad p_{5} = 0,\quad p_{7} = 0,\;{\text{as}}\;\;\eta \to \infty. \hfill \\ \end{gathered} \right.$$

## Numerical results and discussion

In this research, the micropolar 3rd grade fluid flow past an exponentially stretchable sheet is studied. By understanding fluid flow assumptions, the mathematical model is constructed. Introducing the relevant similarity transformations, the system of PDEs is changed into ODEs. Equations. (12)–(15) along with the BC (16) is numerically solved by use of MATLAB. MATLAB software utilizes Runge-Kutta-Fehlberg fourth-fifth order shooting technique to give numerical solutions for BVP. The charactoristics of pertinent parameters involved on the $\phi (\eta )$, $\theta (\eta )$, $f'(\eta )$ and $h(\eta )$ are examined through graphical portrayals.

**Figure 2:**
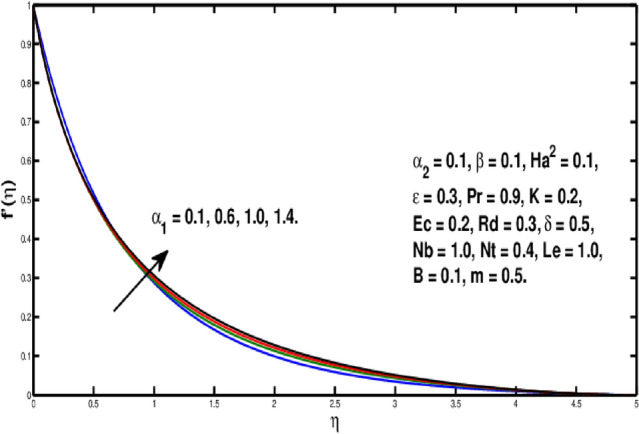
Variation of $\alpha _1$ on $f'(\eta ).$

**Figure 3:**
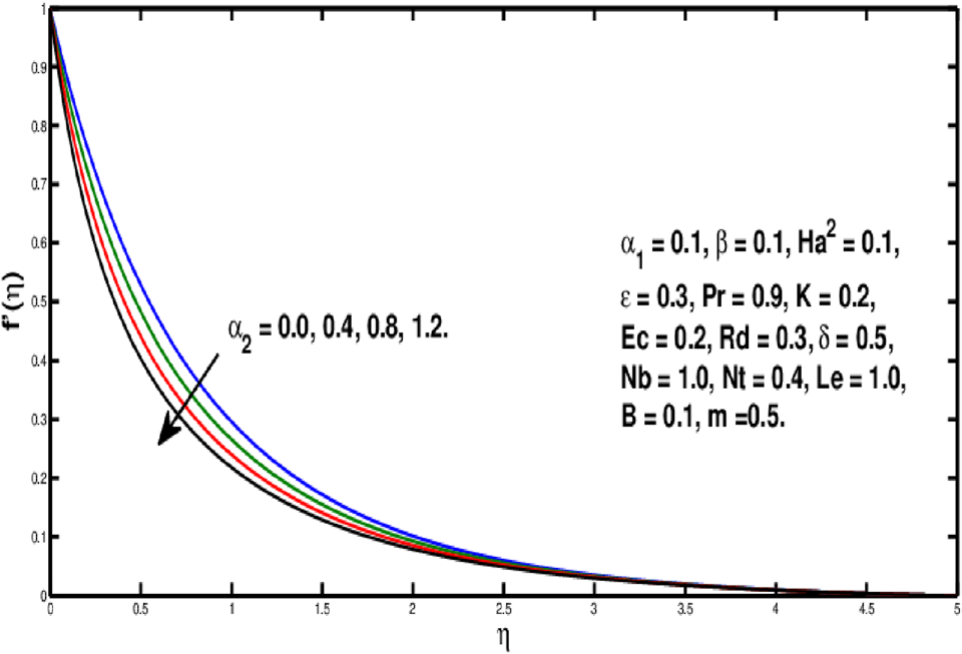
Variation of $\alpha _2$ on $f'(\eta ).$

**Figure 4:**
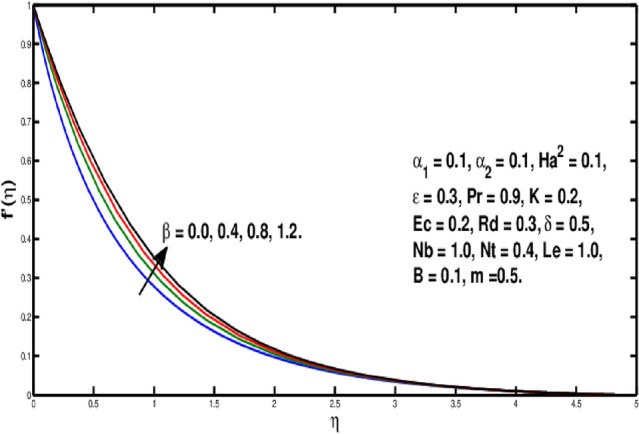
Variation of $\beta$ on $f'(\eta ).$

**Figure 5:**
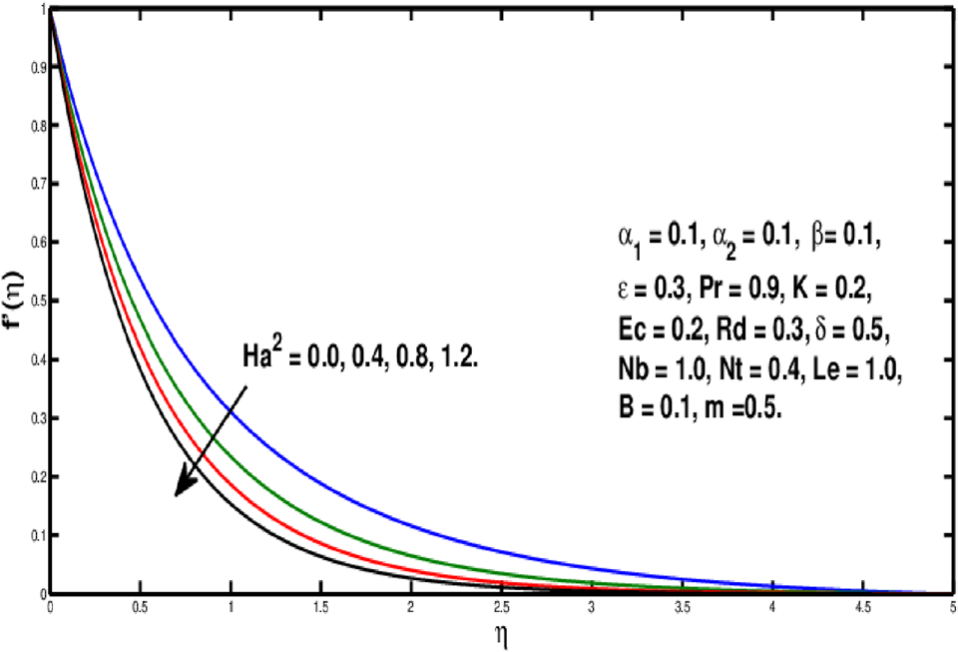
Variation of $Ha^2$ on $f'(\eta ).$

Figure 2 underlines the influence of fluid variable $\alpha _1$ on velocity distribution, which shows the growth of $f'(\eta )$ as rising the parameter $\alpha _1$. Physically, the viscosity of the material lowers if a larger value of $\alpha _1$ is considered because of that force between the adjacent layers reduces, so in this case velocity increases. Figure 3 is the visual portrayal of $\alpha _2$ on velocity. It is visualized that for a higher value of $\alpha _2$, a decline is seen in the curve of velocity distribution. This parameter causes shear-thickening of the fluid and a rise in resistance, that reduces the boundary layer flow, and originates an decrement in the size of the momentum boundary layer width. Figure 4 visualized variation of parameter $\beta$ on velocity field, a rise in the velocity distribution is witnessed for a higher value of $\beta$. Actually $\beta$ is inversely related to the viscosity. For greater $\beta$, viscosity declines and thus velocity rises. Figure 5 shows characteristics of Hartmann number $Ha^2$ on velocity distribution. It seems that velocity falls via larger Hartmann number. This is because, when magnetic field rises then Lorentz forces get stronger. Resistance to fluid flow is now more than before thus velocity is lowered. This process assists in controlling the size of the boundary layer.

**Figure 6:**
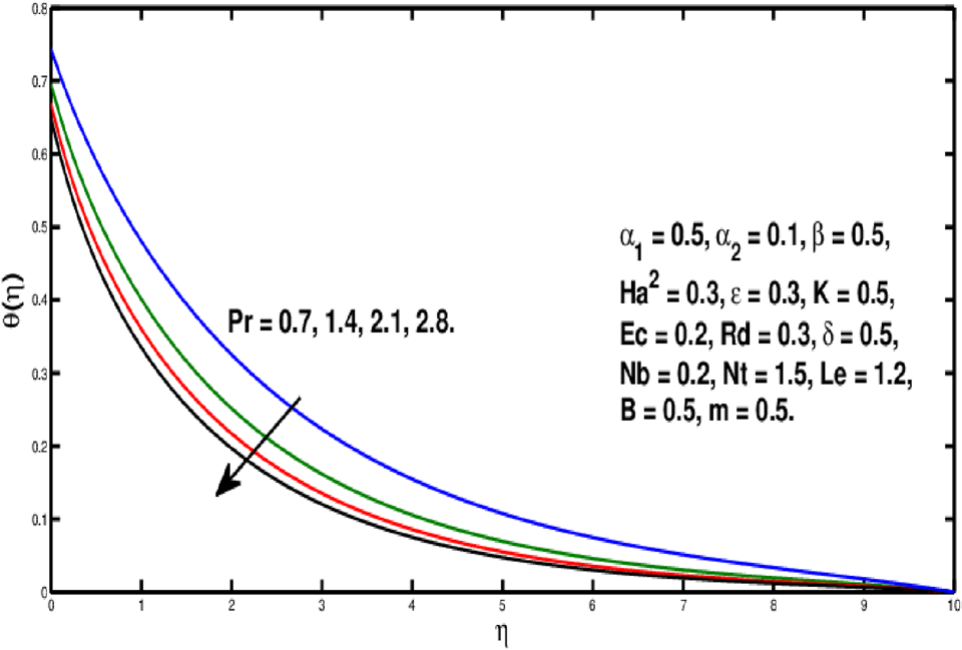
Variation of *Pr* on $\theta (\eta ).$

**Figure 7:**
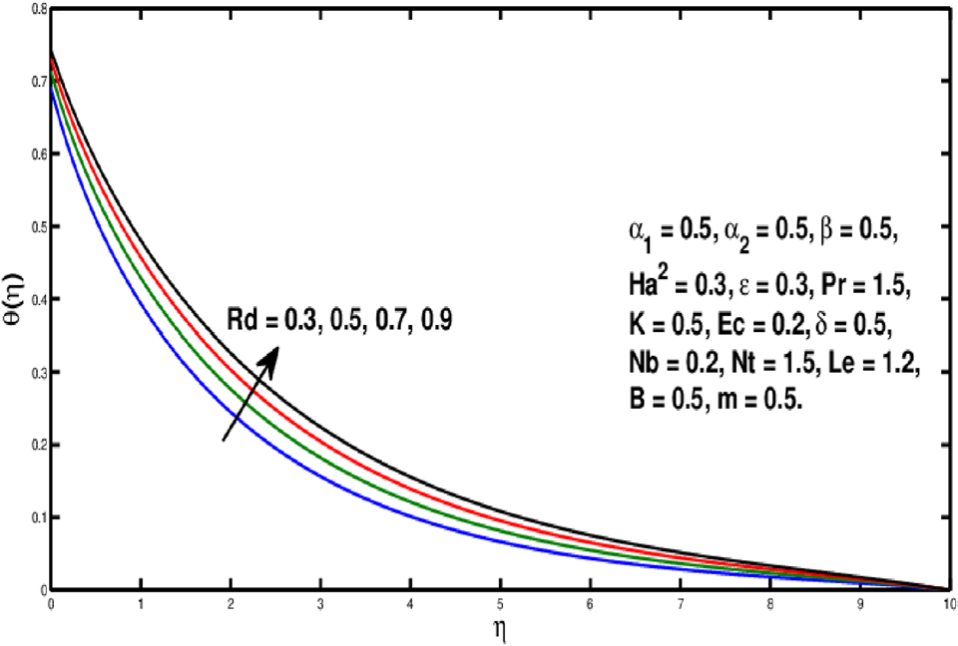
Variation of *Rd* on $\theta (\eta ).$

**Figure 8:**
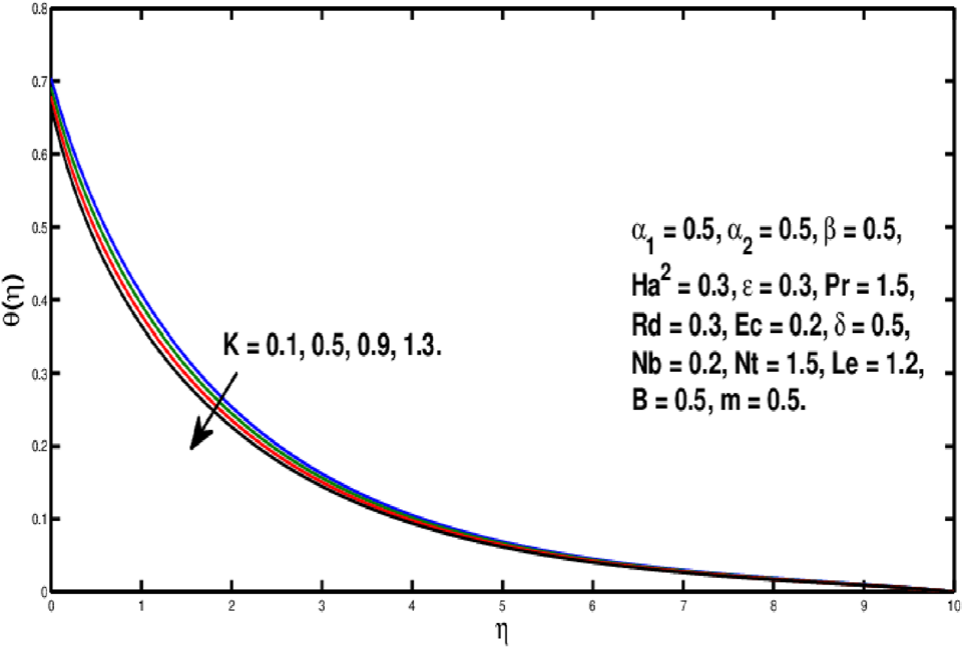
Variation of *K* on $\theta (\eta ).$

**Figure 9:**
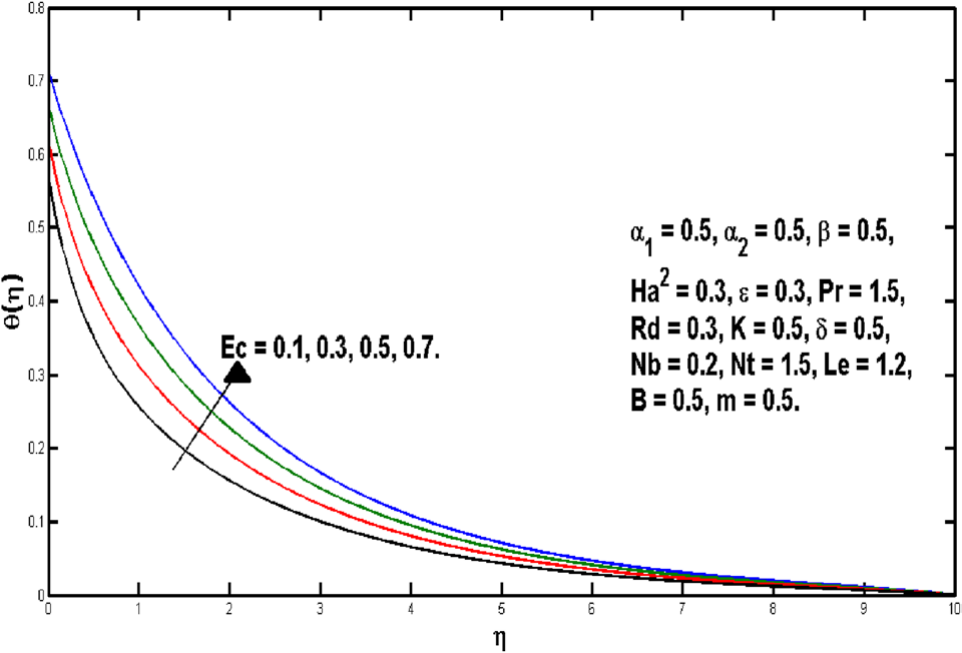
Variation of *Ec* on $\theta (\eta ).$

Figure 6 is describing that temperature of the fluid declines for boosting of *Pr*. Perceptions are that thermal boundary layer thickness appears to decline while the rising upsides of *Pr* are utilized. As a result, when the Prandtl number ascents, pace of thermal conductivity gets higher. In this way, with more noteworthy *Pr*, heat will disperse more rapidly from the sheet. As it’s undeniably true that liquids having prevalent Prandtl number *Pr* will have lesser worth of thermal conduction. Therefore, Prandtl number is utilized to augment the cooling behavior in the flows. Heat exchange for different values of Radiation parameter *Rd* is presented in Fig. 7. A direct relation is witnessed between *Rd* and temperature distribution. It is concluded that with upper values of *Rd* results in enhancement of temperature distribution. In fact, asending value of parameter *Rd* means more heat is transferred to the fluid, that takes temperature distribution to go upward. Figure 8 is visual proof of the effects of micropolar parameter *K* on $\theta (\eta )$, which is an evident that $\theta (\eta )$ is diminishing function of *K*. Figure 9 indicates the temperature $\theta (\eta )$ for different *Ec* values. By boosting *Ec*, an elevation is seen in the temperature distribution. It transfers kinematic energy into inner energy by overcoming viscous fluid tension and converting it to heat. Higher values of the Eckert number enhance the system’s kinetic energy, which raises the temperature. As a result, increased viscous heat dissipation causes both growing heat and rising temperature.

**Figure 10:**
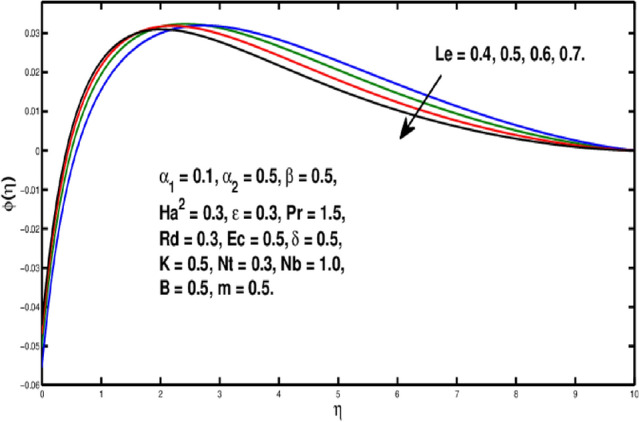
Variation of *Le* on $\phi (\eta ).$

**Figure 11:**
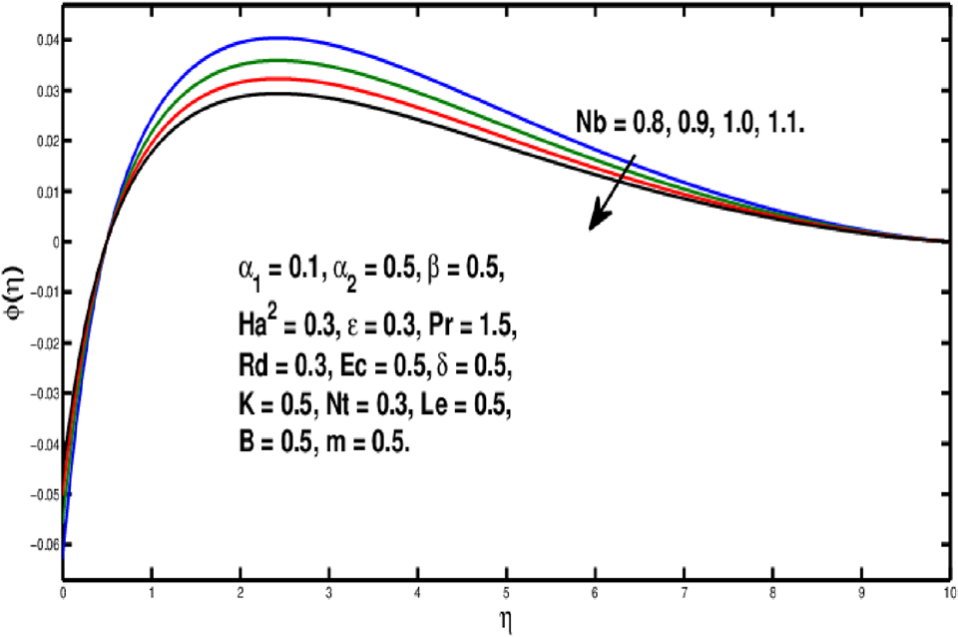
Variation of *Nb* on $\phi (\eta ).$

**Figure 12:**
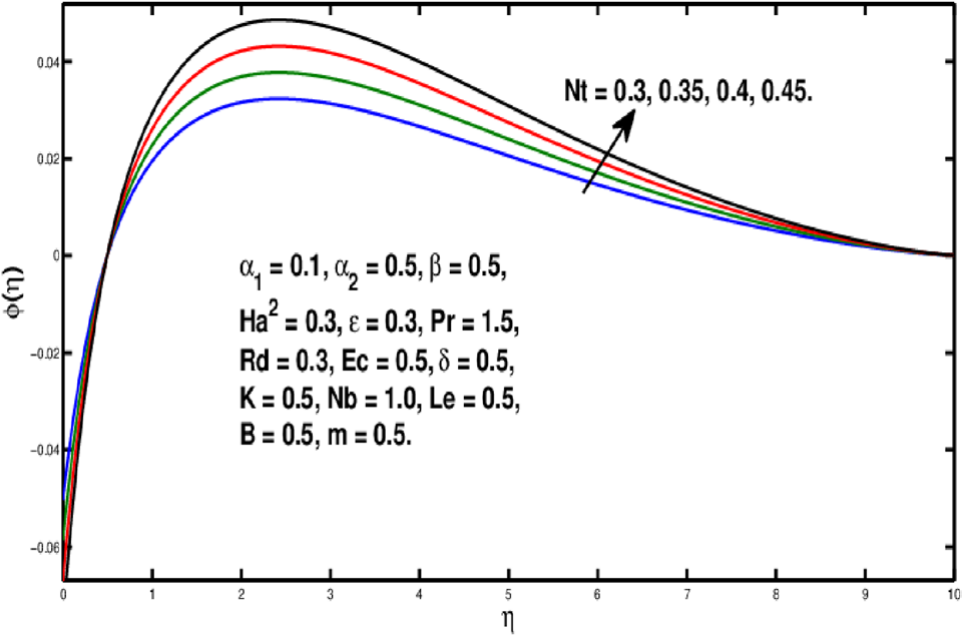
Variation of *Nt* on $\phi (\eta ).$

**Figure 13:**
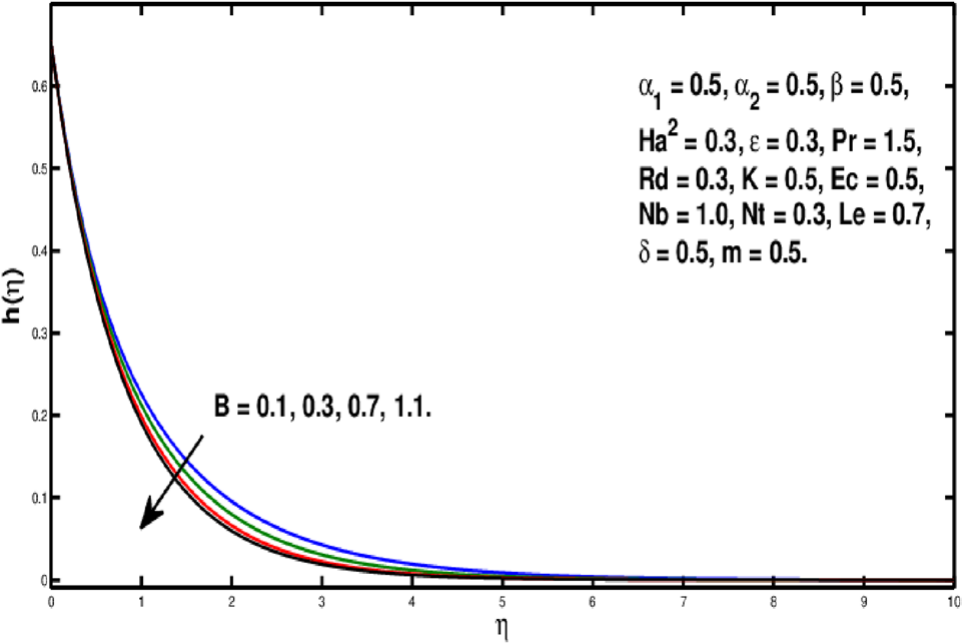
Variation of *B* on $h(\eta ).$

The role of the *Le* on $\phi (\eta )$ is observed in Fig. 10. The Lewis number is stated as the rate of heat-to-mass diffusion coefficient. It is utilized to express the flow of liquid in which heat and momentum transfer occur simultaneously. The concentration distribution becomes steeper when Lewis number is increased. A bigger *Le* implies the lower $D_B$(as it can be seen in Eq. 17 ) which causes a shorter penetration depth for concentration boundary layer. Figure 11 illustrates impact of *Nb* on the concentration field. A rise in Brownian motion variable gives a fall in $\phi (\eta )$ inside boundary layer. Furthermore, the progressing amounts of brownian motion coefficient reduces the micro-mixing of nanoparticles into the fluid’s zone which diminishes the boundary layer thickness of concentration distribution. The consequences of *Nt* on $\phi (\eta )$ is exhibited in Fig. 12. It is seen that dimensionless concentration increases due to thermophoretic parameter. The thermophoresis constant amplifies the thermophoretic force, resulting in the transfer of nanoparticles from warm to cool locations and an increment in nanoparticle volume. In Fig. 13, the impact of Microinertia density parameter *B* is seen on micropolar profile. The behavior of $h(\eta )$ is decreasing for increasing values of *B*.

In Table 1, the impacts of $\alpha _1$, $\alpha _2$, $\beta$, and $Ha^2$ are noticed on the skin-friction coefficient whereas keeping other parameters fixed. Fixed values used are $\epsilon =0.3$, $Pr=1.5$, $K=0.2$, $Ec=0.8$, $\delta =0.5$, $Nt=0.3$, $Rd=0.5$,, $B=1.0$, $\delta _T=0.8$, $m=0.5$,$Nb=1.0$, and $Le=0.7$. It is seen that skin-friction coefficient shows decrement for any larger value of $\alpha _1$, $\alpha _2$, and $Ha^2$ and shows opposite behavior for a larger value of $\beta$. Because the viscosity at the surface of the sheet increases with the evolution of the material constant, the skin friction coefficient decrease. The skin friction coefficient increases as the shear thickening coefficient $\beta$ increases the thickness of the boundary layer. Increasing the Hartmann number $Ha^2$, which refers to a significantly stronger axial magnetic field and an increment in magnetic Lorentz resisting forces in the *x*-direction has a significant impact on the magnitude of primary skin friction coefficient for all axial coordinate values.

**Table 1:** Variation of skin friction coefficient with $\alpha _1$, $\alpha _2$, $\beta$, and $Ha^2$.

$\alpha _1$	$\alpha _2$	$\beta$	$Ha^2$	$\frac{1}{\sqrt{2}}\sqrt{Re_x}Cf_x$
0.1	0.5	0.5	0.3	$-$1.7445
0.3				$-$2.8171
0.5				$-$3.9383
0.7				$-$5.0724
0.1	0.1			$-$1.5369
	0.3			$-$1.6391
	0.5			$-$1.7445
	0.7			$-$1.8523
	0.5	0.1		$-$2.3654
		0.3		$-$1.9467
		0.5		$-$1.7445
		0.7		$-$1.6153
		0.5	0.1	$-$1.6381
			0.2	$-$1.6943
			0.3	$-$1.7445
			0.4	$-$1.7902

In Table 2, the impacts of some of pertinent parameters are observed on the local Nusselt number, while keeping other parameters unchanged as $m=0.5$, $B=1.0$, $\alpha _2=0.5$, $Ha^2=0.3$, $\delta _T=0.8$, $Le=0.7$ $\beta =0.5$, and $\alpha _1=0.1$.

It is seen that Nusselt number gives a rise in its values as a larger value of *Pr*, *K*, *Ec*, *Rd*, $\delta$, and *Nt* is used while Nusselt number shows a decline for higher input of $\epsilon$. The increment in the values of Prandtl number cause a decline in the thermal diffusivity, and hence resist the rise in heat transmission rate at the boundary. Boosting the Prandtl number raises the average Nusselt number at the heated surface. Higher values of the *Rd* improve convective heat transmission which ultimately raises the average Nusselt number.

**Table 2:** Variation of Nusselt number with $\epsilon$,*Pr*,*K*, *Ec*, *Rd*, $\delta$, *Nb*, and *Nt*.

$\epsilon$	*Pr*	*K*	*Ec*	*Rd*	$\delta$	*Nb*	*Nt*	$\sqrt{\frac{2}{X}}\frac{Nu_x}{\sqrt{Re_x}}$
0.2	1.5	0.2	0.8	0.5	0.5	1.0	0.3	0.9230
0.25								0.9205
0.3								0.9180
0.35								0.9155
0.3	0.7							0.7210
	1.1							0.8393
	1.5							0.9180
	2.1							0.9971
	1.5	0.2						0.9180
		0.25						0.9346
		0.3						0.9513
		0.35						0.9680
		0.2	0.2					0.6247
			0.4					0.7211
			0.6					0.8189
			0.8					0.9180
			0.8	0.2				0.8362
				0.3				0.8633
				0.4				0.8907
				0.5				0.9180
				0.5	0.4			0.8822
					0.45			0.9009
					0.5			0.9180
					0.55			0.9337
					0.5	0.9		0.9180
						1.0		0.9180
						1.1		0.9180
						1.2		0.9180
						1.0	0.2	0.9175
							0.3	0.9180
							0.4	0.9185
							0.5	0.9190

**Table 3:** Variation of Sherwood number with *Le*, *Nb*, and *Nt*.

*Le*	*Nb*	*Nt*	$\sqrt{\frac{2}{X}}\frac{Sh_x}{\sqrt{Re_x}}$
0.2	1.0	0.3	$-$0.1650
0.5			$-$0.1652
0.8			$-$0.1653
1.1			$-$0.1654
0.7	0.9		$-$0.1836
	1.0		$-$0.1652
	1.1		$-$0.1502
	1.2		$-$0.1377
	1.0	0.2	$-$0.1101
		0.3	$-$0.1652
		0.4	$-$0.2204
		0.5	$-$0.2757

In Table 3, the behavior of local Sherwood number is observed for some of physical parameters involved. The following parameters are taken as unchanged in this course $\alpha _1=0.1$, $\delta =0.5$,$B=1.0$, $\alpha _2=0.5$, $Pr=1.5$, $Ha^2=0.3$, $\beta =0.5$, $\delta _T=0.8$,$\epsilon =0.3$, $Pr=1.5$, $K=0.2$, $Ec=0.8$, $Rd=0.5$, and $m=0.5$. It is seen that Sherwood number shows increment for bigger values of *Le* and thermophoretic parameter *Nt* and decrement for higher value of Brownian motion number *Nb*.

## Concluding remarks

Third-grade micropolar fluid flow is analyzed in this research article. An ODEs system is generated from the PDEs system by a similarity transformation and then analytically solved by the fourth-fifth order Runge-Kutta-Fehlberg strategy. Several graphs are presented for the temperature, velocity, concentration, and micropolar fields to observe the impact of different parameters on them. The impact of various parameters on Nusselt number, skin-friction coefficient, and Sherwood number is also concluded. The main findings are

- Velocity distribution $f'(\eta )$ boosts with $\alpha _1$ $\beta$, while it has opposite behavior for $\alpha _2$ and Hartmann number $Ha^2$.
- Temperature distribution $\theta (\eta )$ presents an increasing behavior for Radiation parameter *Rd*, and Eckert number *Ec*, while, the opposite behavior for *Pr*, and *K*.
- Concentration distribution $\phi (\eta )$ shows diminishing behavior as we put a higher value of Brownian motion number *Nb* and *Le*. On the contrary, this has the opposite behavior for *Nt*.
- Micropolar distribution $h(\eta )$ displays an opposite relation with microinertia density parameter *B*.
- Skin-friction coefficient goes on increasing for higher values of parameters $\alpha _1$, $\alpha _2$, and $Ha^2$ and shows opposite behavior for bigger values of $\beta$.
- Nusselt number shows an increasing behavior as larger values of parameters *Pr*, *K*, *Ec*, *Rd*, $\delta$, and *Nt* are used, while Nusselt number depicts a decline for higher input of parameter $\epsilon$.
- Sherwood number shows increment for bigger values of parameters *Le* and *Nt* and decrement for the higher value of parameter *Nb*.

## Data Availability

All data generated or analyzed during this study are included in this article.
