# Author Correction: Insight into the heat transfer of third-grade micropolar fluid over an exponentially stretched surface

**DOI:** 10.1038/s41598-022-23245-2

**Published:** 2022-11-02

**Authors:** Kamel Guedri, N. Ameer Ahammad, Sohail Nadeem, ElSayed M. Tag-ElDin, Aziz Ullah Awan, Mansour F. Yassen

**Affiliations:** 1 Mechanical Engineering Department, College of Engineering and Islamic Architecture, Umm Al-Qura University, P.O. Box 5555, Makkah, 21955 Saudi Arabia; 2 Department of Mathematics, Faculty of Science, University of Tabuk, P.O. Box 741, Tabuk, 71491 Saudi Arabia; 3 Department of Mathematics, Quaid-i-Azam University, Islamabad, 45320 Pakistan; 4 Faculty of Engineering and Technology, Future University in Egypt, New Cairo, 11835 Egypt; 5 Department of Mathematics, University of the Punjab, Lahore, 54590 Pakistan; 6 Department of Mathematics, College of Science and Humanities in Al-Aflaj, Prince Sattam Bin Abdulaziz University, Al-Aflaj, 11912 Saudi Arabia; 7 Department of Mathematics, Faculty of Science, Damietta University, New Damietta, 34517 Damietta Egypt

Correction to: *Scientific Reports* 10.1038/s41598-022-19124-5, published online 16 September 2022

The original version of this Article contained an error in the Acknowledgements section. The last digit of the Grant Code was incorrect.

“The authors would like to thank the Deanship of Scientific Research at Umm Al-Qura University for supporting this work by Grant Code: 22UQU4331317DSR83.”

now reads:

“The authors would like to thank the Deanship of Scientific Research at Umm Al-Qura University for supporting this work by Grant Code: 22UQU4331317DSR81.”

The original Article has been corrected.

